# In Vitro Anticancer Screening and Preliminary Mechanistic Study of A-Ring Substituted Anthraquinone Derivatives

**DOI:** 10.3390/cells11010168

**Published:** 2022-01-05

**Authors:** Ibrahim Morgan, Ludger A. Wessjohann, Goran N. Kaluđerović

**Affiliations:** 1 Department of Bioorganic Chemistry, Leibniz Institute of Plant Biochemistry, Weinberg 3, 06120 Halle (Saale), Germany; ibrahim.morgan@ipb-halle.de; 2 Department of Engineering and Natural Sciences, University of Applied Sciences Merseburg, Eberhard-Leibnitz-Straße 2, 06217 Merseburg, Germany

**Keywords:** anthraquinone, cytotoxicity, apoptosis, cell cycle, caspase, autophagy, proliferation, topoisomerase

## Abstract

Anthraquinone derivatives exhibit various biological activities, e.g., antifungal, antibacterial and in vitro antiviral activities. They are naturally produced in many fungal and plant families such as Rhamnaceae or Fabaceae. Furthermore, they were found to have anticancer activity, exemplified by mitoxantrone and pixantrone, and many are well known redox-active compounds. In this study, various nature inspired synthetic anthraquinone derivatives were tested against colon, prostate, liver and cervical cancer cell lines. Most of the compounds exhibit anticancer effects against all cell lines, therefore the compounds were further studied to determine their IC_50_-values. Of these compounds, 1,4-bis(benzyloxy)-2,3-bis(hydroxymethyl)anthracene-9,10-dione (**4**) exhibited the highest cytotoxicity against PC3 cells and was chosen for a deeper look into its mechanism of action. Based on flow cytometry, the compound was proven to induce apoptosis through the activation of caspases and to demolish the ROS/RNS and NO equilibrium in the PC3 cell line. It trapped cells in the G2/M phase. Western blotting was performed for several proteins related to the effects observed. Compound **4** enhanced the production of PARP and caspase-3. Moreover, it activated the conversion of LC3A/B-I to LC3A/B-II showing that also autophagy plays a role in its mechanism of action, and it caused the phosphorylation of p70 s6 kinase.

## 1. Introduction

Cancer is one of the most common and threatening human diseases. In 2018, it was estimated that 18.1 million persons were diagnosed with cancer worldwide. It was also estimated that cancer caused around 9.6 million cases of death [1]. According to the federal statistics office (2017), cancer is the second most leading cause of death in Germany, which was estimated to 25% of all cases of death [2].

Several methods are already established for the treatment of cancer, such as chemotherapy, hormonal therapy, immunotherapy, surgery and radiotherapy [3,4]. Chemotherapy is considered the mainstay of cancer treatment combined with either radiotherapy or surgery. This therapy includes various groups of agents, each has a specific mode of action such as alkylating agents, metal-based agents, antimetabolites, topoisomerase inhibitors and tubulin binding agents, etc. [5]. Several new chemotherapeutic agents were found to induce autophagic cell death (ACD) through the accumulation of autophagosomes and autolysosomes in the cytoplasm [6]. The malfunctioning cellular components are engulfed by autophagosomes which promote their fusion with autolysosomes and the release of digestive enzymes, leading to the digestion and recycling of the engulfed material [7,8,9]. However, autophagy can also be activated as a cytoprotective mechanism against chemotherapeutic agents. In which, autophagy counteracts the action of apoptosis through the elimination of the damaged organelles and proteins leading to the production of energy which could be utilized for the survival of the cell [10]. The role of autophagy can be determined by the use of autophagy inhibiting agents, e.g., 3-methyladenine, and studying its impact on cell survival [11]. 

**Figure 1:**
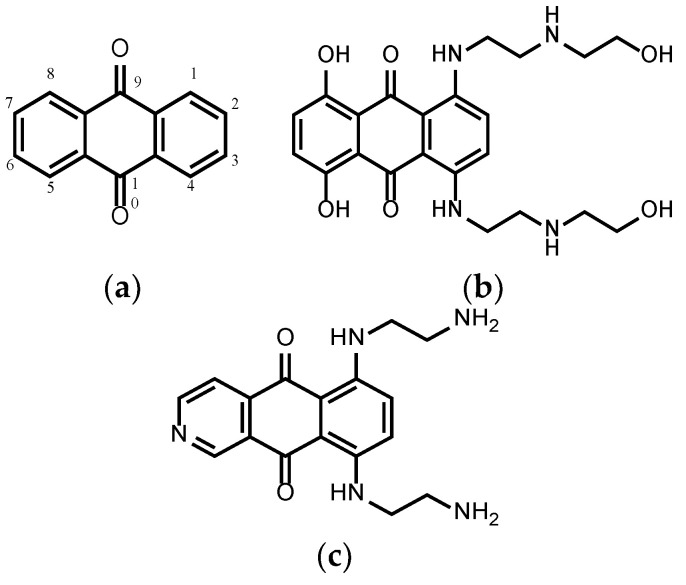
(**a**) Anthraquinone core, (**b**) mitoxantrone and (**c**) pixantrone.

Anthraquinone derivatives are a group of aromatic compounds that have anthraquinone (Figure 1a) as main structural core. They are widely used nowadays in the dye industry [12], paper industry and for the synthesis of H_2_O_2_ [13]. This group of compounds exists naturally in different plant families such as Rhamnaceae [14], Fabaceae [15], Hypericaceae [16] and, moreover, they can be found in insects [17] and fungi [18]. Furthermore, anthraquinones can be synthesized chemically, e.g., by a Friedel–Crafts acylation [19]. Nevertheless, in medicinal chemistry, anthraquinones are not always popular as they can belong to so-called PAINS and frequent hitters [20], as many are fluorescent, redox-active or generally protein binding like other phenolics too [21]. However, the fact that nature uses these compounds frequently to achieve effects should teach us to look deeper into their usefulness and mechanisms.

Anthraquinone derivatives were proven to possess antifungal, antibacterial [22,23,24,25,26] and in vitro antiviral activity [27,28,29]. Anthraquinones are also used in the treatment of other diseases and already available in the market [30]. Senna glycosides are previously proven to have a stimulant laxative effect [15,31,32]. Scutianthraquinones A, B and C are naturally occurring anthraquinones extracted from the bark of *Scutia myrtina*. They are antiparasitic agents against *Plasmodium falciparum*, one of the protozoans causing malaria [33]. Moreover, anthraquinones were proven to act as antioxidants and cholinesterase inhibitors which decrease the degeneration in Alzheimer’s patients [34].

Some anthraquinones are used for treating cancers [35,36]. Mitoxantrone (Figure 1b) is an anthraquinone compound which is used for the treatment of ALL (acute lymphoblastic leukemia) and it was proven to improve the outcome in children diagnosed with relapsed ALL compared to idarubicin [37]. Pixantrone (Figure 1c) is an azaanthraquinone that has antineoplastic activity, which has completed phase III clinical trials. Pixantrone showed a reduction in cardiotoxicity compared to anthracycline [38,39].

**Figure 2:**
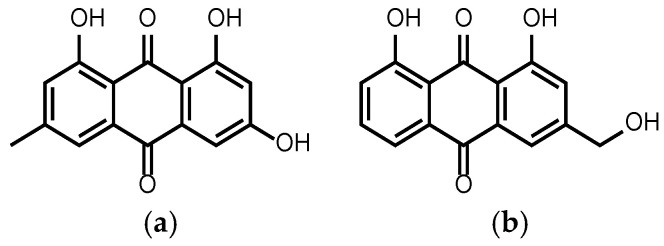
(**a**) Emodin and (**b**) aloe-emodin.

Various anthraquinone derivatives are now under further investigation [40]. Emodin (Figure 2a) is a natural compound extracted, e.g., from *Rheum palmatum*. It shows activity against various cancer types or enhancement of the anticancer activity of other anticancer agents. The anticancer activity of ATRA (all-trans retinoic acid) against NB4 cells (acute myeloid leukemia cancer cells) can be enhanced when combined with emodin and the combination leads to differentiation of the cancer cells. It also increases the sensitivity of ATRA-resistant cells (MR2, NB4-derived ATRA-resistant cancer cells) to ATRA. Emodin induces apoptosis and growth inhibition of AML (acute myeloid leukemia) cells through the activation of caspase-9, caspase-3, PARP (poly-ADP-ribose-polymerase), cleavage and decrease of the expression of antiapoptotic factors such as *Bcl-2* (B-cell lymphoma 2). Moreover, emodin was proven to have an inhibitory effect on PTK (protein tyrosine kinase) [41]. Due to this effect, emodin decreases the *HER-2*/*neu* tyrosine kinase (Receptor tyrosine-protein kinase *erbB-2*) which is involved in the development of chemoresistance. The combination with chemotherapeutic agents provided effective anticancer options against *HER-2*/*neu* overexpressing cells [42]. Furthermore, emodin was proven to be effective in prostate cancer especially when combined with gemcitabine. The combination caused a significant reduction in the tumor volumes by the down-regulation of several proteins such as survivin or XIAP (X-linked inhibitor of apoptosis protein) [43]. Another example for the anthraquinone compounds which are under anticancer investigation is aloe-emodin. Aloe-emodin (Figure 2b) was proven to induce autophagic death of C6 (glioma) cells and the differentiation of the surviving cells to normal astrocytes through the inhibition of ERK1/2 (extracellular receptor kinase) [44]. These examples show that this family of compounds not only can induce cell death but also has the ability to differentiate cancer cells back to normal, a property which renders them highly relevant for a deeper study. 

The present study aims to determine the anticancer activity of 29 different anthraquinone derivatives and compares these to emodin and anthraquinone as references, specifically all structural derivatives containing an anthraquinone core available in sufficient quantity and purity from the collection of the Leibniz Institute of Plant Biochemistry, against PC3 (focus cell line), HT-29, HeLa and HepG2 cell lines.

The anthraquinone derivative with the lowest IC_50_ will be studied in detail to determine its mechanism of action on PC3 cells. Namely, cell cycle distribution, cellular proliferation, autophagy induction, the formation of ROS/RNS (reactive oxygen/nitrogen species) upon treatment with the most active compound will be evaluated. Moreover, western blot analysis will contribute to understand the effect of the lead compound on the expression of different proteins in the cancer cells treated with it.

## 2. Materials and Methods

### 2.1. Chemicals and Cell Lines

Carboxyfluorescein succinimidyl ester (CFSE) and dihydrorhodamine 123 (DHR) were bought from BD Horizon, Franklin Lakes, NJ, USA. Phosphate buffered saline (PBS), RPMI 1640 and Trypsin EDTA were from Capricorn Scientific, Ebsdorfergrund, Germany. β-mercaptoethanol was from Bio-Rad, Hercules, CA, USA. Anti-rabbit IgG, HRP- linked Antibody, p38 MAPK rabbit Ab, α/β- Tubulin rabbit Ab, p70 S6 Kinase rabbit Ab, Caspase-3 rabbit Ab, P-p38 MAPK (T100/Y182) rabbit Ab, P-p70 S6 Kinase (T389) rabbit Ab, PARP rabbit Ab and LC3A/B (D3U4C) XP^®^ Rabbit mAb were purchased from Cell Signaling Technology, Danvers, MA, USA. Dimethyl sulfoxide (DMSO) was bought from Duchefa Biochemie, Haarlem, The Netherlands. ECL Prime Western Blotting System was supplied by GE Healthcare, Chicago, IL, USA. Annexin V/propidium iodide (AnnV/PI), PAGE Ruler, EDTA Solution, Trypan blue and Halt Protease Inhibitor Cocktail were obtained from Thermofisher Scientific, Waltham, MA, USA. Ethanol, Na_2_HPO_4_, NaH_2_PO_4_ and BSA were bought from Merck, Kenilworth, NJ, USA. Apostat was purchased from R & D systems, Minneapolis, MN, USA. Digitonin was from Riedel De Haen, Seelze, Germany. Acetic acid, ammonium persulfate (APS), fetal calf serum (FCS), Glycerol, Glycine, Methanol, NaOH, penicillin/streptomycin, ROTI®Quant, tetramethylethylenediamine (TEMED) and tris(hydroxymethyl)aminomethane (TRIS) were from Roth, Karlsruhe, Germany. Acrylamide/bisacrylamide was bought from Serva, Heidelberg, Germany. Finally, acridine orange (AO), bromophenol blue, crystal violet (CV), 4′,6-diamidino-2-phenylindole (DAPI), 3-(4,5-dimethylthiazol-2-yl)-2,5-diphenyltetrazolium bromide (MTT), paraformaldehyde (PFA), Triton X-100 and Tween-20 were from Sigma Aldrich, St. Louis, MO, USA.

The compound collection of the Leibniz Institute of Plant Biochemistry (IPB) was screened for the anthraquinone derivatives available in sufficient amount, purity (>98%) and with defined structure. 33 compounds resulted, including 2 commercial anthraquinones (**30, 31**) and 2 reference ones (**32, 33**). The compounds are illustrated in Table S1. The selected library compounds to our best knowledge were not studied for antitumor activity. All investigated compounds are already described in literature [45,46,47,48,49], purity and structure were confirmed using ^1^H and ^13^C NMR spectroscopy as well as ESI-MS. ^1^H NMR (CDCl_3_ or DMSO-*d_6_*, 400 MHz) and ^13^C NMR (CDCl_3_ or DMSO-*d_6_*, 100 MHz) spectra were recorded in CDCl_3_ or DMSO-*d_6_* solutions on a Varian Mercury 400 spectrometer. Chemical shifts (*δ*) were determined in ppm relative to TMS (^1^H NMR) and either residual CDCl_3_ or DMSO-*d_6_* signal (^13^C NMR). ESI-MS was recorded on a Finnigan TSQ 7000, LC-Tech Ultra Plus pumps, Linear UV/V is 200 detector, SepserveUltrasep ES RP-18 5 μm 1 × 100 mm column, and flow 70 μL/min. ^1^H and ^13^C NMR spectroscopy confirmed the structure of selected compounds.

To determine the cytotoxic effect of the anthraquinone derivatives, several cancer cell lines of different origins were used. Prostate, colon, cervix and liver cancer cell lines (PC3, HT-29, HeLa and HepG2; respectively) were included. These cell lines were from the cell line stock of the Leibniz Institute of Plant Biochemistry. The cells were grown in RPMI 1640 medium supplemented with 10% FCS and 1% penicillin/streptomycin at 37 °C and 5% CO_2_. Cells were seeded at 1.5 × 10^3^ cells/well in 96-well plates for viability determination and 1 × 10^5^ cells/well in 6-well plates for flow cytometry and western blotting.

### 2.2. Cell Viability

To identify the compounds with sufficient anticancer activities, the four cell lines were treated with 0.01 and 10 µM of anthraquinone compounds for 48 h. The compounds which showed anticancer activity were further analyzed to determine their IC_50_, the cells were treated with the compounds using a concentration series (100, 50, 25, 12.5, 6.25, 3.13, 1.56 µM) for 48 h. The cell viability was determined using CV and MTT assay. For the CV assay, cells were fixed using 4% paraformaldehyde for 10 min at room temperature, stained with 1% CV solution for 15 min followed by washing using double distilled (dd) H_2_O and dried overnight at RT. Finally, 33% acetic acid was used to dissolve the dye. Regarding the MTT assay, cells were incubated in MTT solution (0.5 mg/mL) for 20 min then the developed formazan was dissolved using DMSO. For both assays, plates were measured using a microplate reader (Spectramax, Molecular Devices, San Jose, CA, USA) at 570 nm with a background wavelength of 670 nm. All results are normalized using cells treated with complete medium and 125 µM of digitonin representing 0 and 100% respectively and presented as percentage. The IC_50_ was calculated using nonlinear regression and four parametric methodology of log concentration versus cell viability [50,51,52].

### 2.3. Flow Cytometry Analysis

#### 2.3.1. Cell Cycle Analysis

The PC3 cells were prepared in a 6 well plate and treated with IC_50_ and 2 × IC_50_ of compound **4** (4.65 and 9.30 µM) and incubated for 48 h at 37 °C under 5% CO_2_. Afterwards, the cells were fixed in 70% ethanol overnight at 2 °C and then, stained with 1 µg/mL of DAPI [53] at RT for 10 min. At last, the cells were analyzed by flow cytometry (FACS Aria III, BD Biosciences, Franklin Lakes, NJ, USA).

#### 2.3.2. Apoptosis, Caspase Production and Autophagy Analysis

The PC3 cells were prepared in a 6 well plate, treated with IC_50_ and 2 × IC_50_ of compound **4** and incubated for 48 h at 37 °C under 5% CO_2_. After the incubation, cells were stained either by AnnV/PI (5 µL of AnnV, 2 µL of PI in 100 µL) PBS to determine apoptosis or by Apostat (1 µL of Apostat, 5% FCS in 100 µL of PBS) to study caspases production. The procedure was carried out according to the manufacturer’s instructions. For autophagy analysis, cells were incubated for 15 min at 37 °C in 1 µg/mL of AO in PBS, washed and resuspended in PBS. The cells were analyzed by flow cytometry [54].

#### 2.3.3. Cell Division Analysis

PC3 cells were stained with 1 µM of carboxyfluorescein succinimidyl ester (CFSE) for 10 min at 37 °C, and then exposed to IC_50_ and 2 × IC_50_ of compound **4** for 48 h. At the end of cultivation, cells were washed, trypsinized and analyzed with a flow cytometry [54].

#### 2.3.4. Investigation of ROS/RNS and NO Production

Reactive oxygen and nitrogen species are produced in response to environmental stress and tissue injury. They were proven to be crucial for the regulation of cell death mechanisms as apoptosis and the homeostasis between autophagy and apoptosis [55]. For the detection of ROS/RNS, PC3 cells were stained with 1 µM of DHR solution for 10 min, afterwards the cells were treated with IC_50_ and 2 × IC_50_ of compound **4** for 48 h. After 48 h, cells were trypsinized, washed with PBS and then analyzed with flow cytometry.

Moreover, NO was also confirmed to be a regulator of autophagy and apoptosis. NO production leads to the inhibition of BcL-2 phosphorylation and hence reducing its antiapoptotic effect [56]. The reduced phosphorylation leads to increase in the interaction between BcL-2/Beclin-1 causing autophagy inhibition [57]. Therefore, NO production was studied, PC3 cells were treated with same concentrations of compound **4**. After 48 h, the cells were incubated with 5 µM DAF-FM diacetate in 10% FCS RPMI 1640 for 1 h at 37 °C and the stain was deactivated by incubation for 15 min with serum free medium. The cells were then detached, resuspended in PBS and analyzed by flow cytometry [58,59].

### 2.4. Western Blot Analysis

PC3 cells were cultivated with an IC_50_ dose of compound **4** for 2 h, 6 h, 12 h, 24 h and 48 h. Protein lysis buffer (62.5 mM Tris–HCl (pH 6.8), 2% (*w*/*v*) SDS, 10% glycerol, and 50 mM dithiothreitol) was used to lysis the cells. The electrical separation of the isolated proteins was performed using 12% SDS-polyacrylamide gels where a PageRuler prestained ladder was used as protein molecular weight marker. The proteins were electrically transferred to nitrocellulose membranes by a western blot system (Owl HEP-1, Thermofisher Scientific, Waltham, MA, USA). The membranes were blocked with 5% (*w*/*v*) BSA in PBS with 0.1% Tween 20 for 1 h at RT. Afterwards, Blots were incubated over night at 4 °C with p38 MAPK rabbit Ab, α/β- Tubulin rabbit Ab, p70 S6 Kinase rabbit Ab, Caspase-3 rabbit Ab, P-p38 MAPK (T100/Y182) rabbit Ab, P-p70 S6 Kinase (T389) rabbit Ab or PARP rabbit Ab, LC3A/B (D3U4C) XP^®^ Rabbit mAb. As a secondary antibody Anti-rabbit IgG, HRP- linked antibody was used. Bands were visualized using an ECL Prime Western Blotting System.

### 2.5. Autophagy Inhibitor Assay

PC3 cells were seeded in a 96-well plate as illustrated in Section 2.2. Cells were treated for 6 h with an autophagy inhibitor 3-methyl adenine (3-MA) of concentration 500 µM in completed medium. Afterwards, the cells were washed with PBS and treated with IC_50_ of compound **4** (4.65 µM). The cells were incubated for 48 h and then the viability was determined by MTT and CV assay (Section 2.2) [11,60].

### 2.6. Topoisomerase I Assay

The experiment was performed to determine the impact of compound **4** on topoisomerase I (topoI) using topoisomerase detection kit and topoI enzyme. The reaction mixture consists of 2 µL of 10× TGS, 500 ng of pHOT1 DNA, 1 U of topoI and different concentration of compound **4** (0–50 µM). The reaction mixture volume was filled to 20 µL using ddH_2_O and incubated for 30 min in 37 °C. Samples were loaded in 1% agarose gel and the gel run was performed using 60 V for 2 h. The bands were visualized under UV light using 0.5 µg/mL of ethidium bromide. The obtained photo was processed by image J to determine each band intensity.

## 3. Results

### 3.1. Impact of Anthraquinone Compounds on the Cell Viability

The fast screening was performed in order to determine the anthraquinone derivatives which showed a significant reduction in the cell viabilities against the four cell lines PC3, HT-29, HeLa and HepG2. Most of the compounds showed a remarkable reduction in the cell viability of at least one of the four cell lines. Therefore, all the compounds were further analyzed to determine their IC_50_ values. The effects of the compounds on PC3, HT-29, HeLa and HepG2 are displayed in the Supplementary Information (Figures S1–S4).

Since all compounds were active in the fast-screening assay, the IC_50_ determination was carried out to determine the cytotoxic potential of all compounds. The obtained viabilities were used to plot the dose-dependent response (Figures S5–S12). The IC_50_ for each compound was determined and the values are shown in Table S1.

The determination of the cell viabilities by MTT assays was not performed for compound **29** because it reacted with formazan producing a blue color which interferes with the absorbance. 

**Figure 3:**
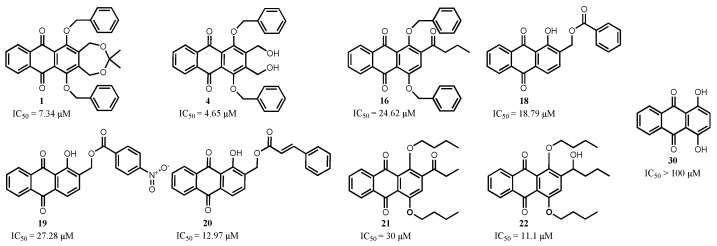
Structural variations of the 1-hydroxyl-anthraquinone core structure and IC_50_ values against prostate cancer cells (PC3 in CV assay).

Compound **4** showed the lowest IC_50_ of 4.65 µM against the PC3 cell line, therefore it was selected for further analysis to determine its mode of action by flow cytometry and western blot analysis. The structure of compound **4** is given in Figure 3.

### 3.2. Compound 4 Induces Trappment of PC3 Cells in G2/M Phase as well as Activation of Caspase Dependent Apoptosis

**Figure 4:**
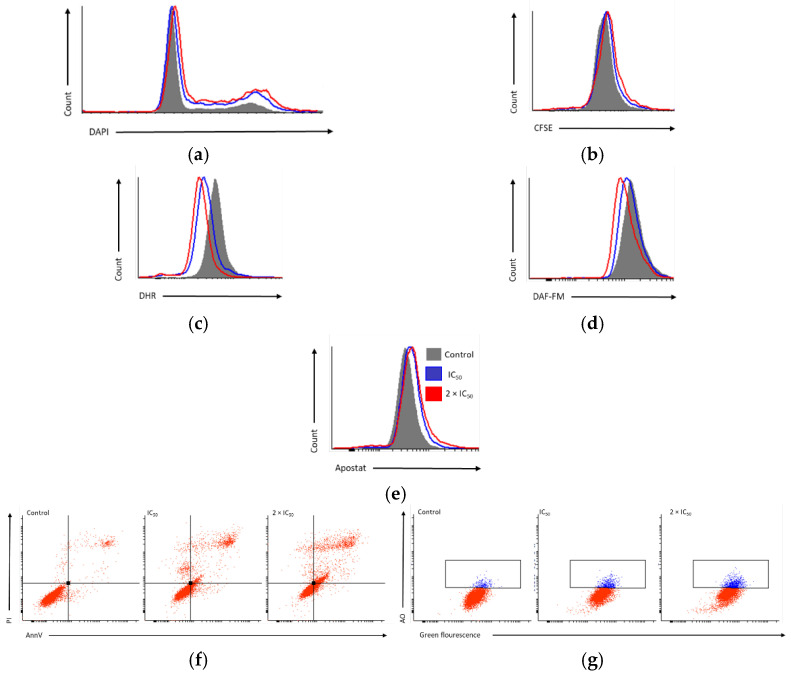
Representative histograms and dot plots for the impact of compound **4** using IC_50_ and 2 × IC_50_ for 48 h against PC3 cell line on (**a**) the cell distribution in G0/G1, S and G2/M phases using DAPI stain; (**b**) the inhibition of PC3 cells proliferation, in which cells were stained using CFSE reagent, later treated with the lead compound; (**c**) the induction of ROS/RNS production, determined with DHR assay; (**d**) NO production, using DAF-FM dye; (**e**) the induction of Caspases production, using Apostat staining kit; (**f**) the induction of apoptosis, using AnnV/PI double staining; (**g**) induction of autophagy, with acridine orange assay. For CFSE, DHR, DAF-FM, AnnV, and Apostat stains the fluorescence was analyzed using excitation 488 ± 20 nm and emission of 530 ± 30 nm (showed on the *x*-axis). For DAPI channel, the fluorescence was analyzed using excitation of 375 ± 20 nm and emission of 450 ± 20 nm (presented on *x*-axis). For AO and PI dyes, the fluorescence was analyzed using excitation and emission of 488 ± 20/695 ± 40 or 561 ± 20/610 ± 20 nm, respectively, (presented on the *y*-axis).

**Figure 5:**
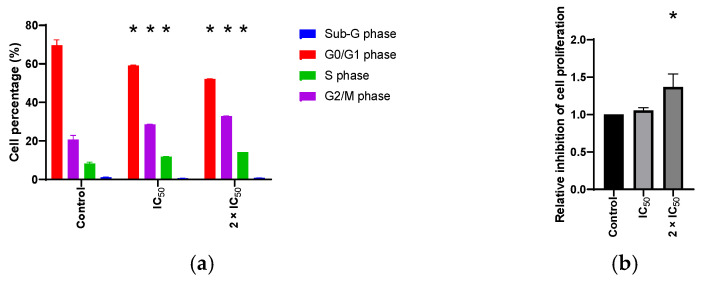

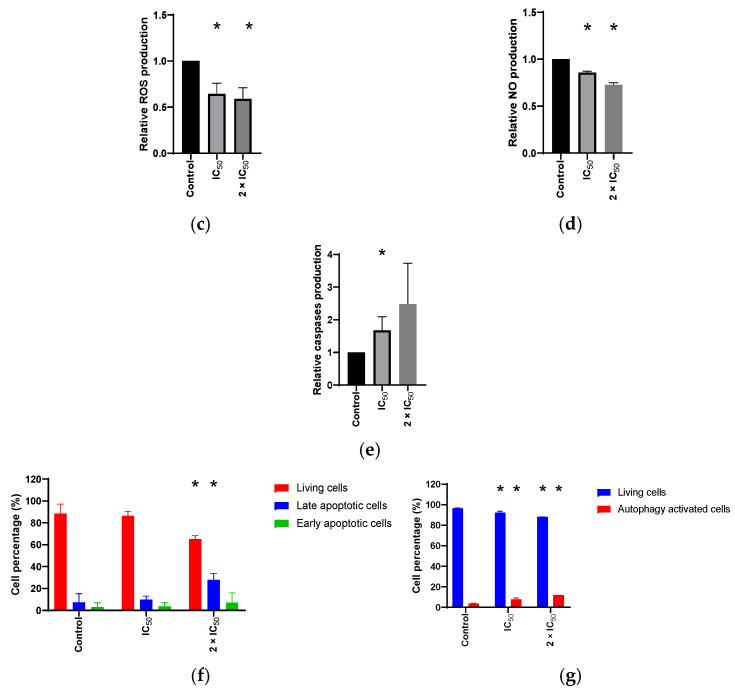
Bar graphs representing the impact of compound **4** using IC_50_ and 2 × IC_50_ for 48 h against PC3 cell line on (**a**) the cell distribution in G0/G1, S and G2/M phases; (**b**) the inhibition of PC3 cells proliferation; (**c**) the induction of ROS/RNS production; (**d**) NO production; (**e**) the induction of caspases production; (**f**) the induction of apoptosis; (**g**) induction of autophagy. Data is normalized to the corresponding value in the untreated sample. Bars represent the mean values ± standard deviation calculated from three independent measurements. * *p* < 0.05 compared to the untreated control cells.

The influence of compound **4** on the cell cycle distribution, proliferation potential and regulation of ROS/RNS as well as NO species was evaluated in PC3 cells. Compound **4** caused the entrapment of PC3 cells in the G2/M phase and slightly in the S phase, preventing the cells from entering the G1 phase (Figure 4a and Figure 5a), caused an insignificant reduction of cell proliferation (Figure 4b and Figure 5b) and a reduction of ROS/RNS (Figure 4c and Figure 5c) and NO production (Figure 4d and Figure 5d).

Furthermore, activation of apoptosis (Figure 4f and Figure 5f), involvement of caspases (Figure 4e and Figure 5e), and induction of autophagy (Figure 4g and Figure 5g) after treatment of PC3 cells was investigated. Compound **4** induces apoptosis by upregulation of caspases. Additionally, slightly elevated amounts of autophagic vesicles were detected in treated PC3 cells.

### 3.3. The Effect of Compound 4 on Apoptosis and Autophagy Related Proteins in PC3 Cell Line

**Figure 6:**
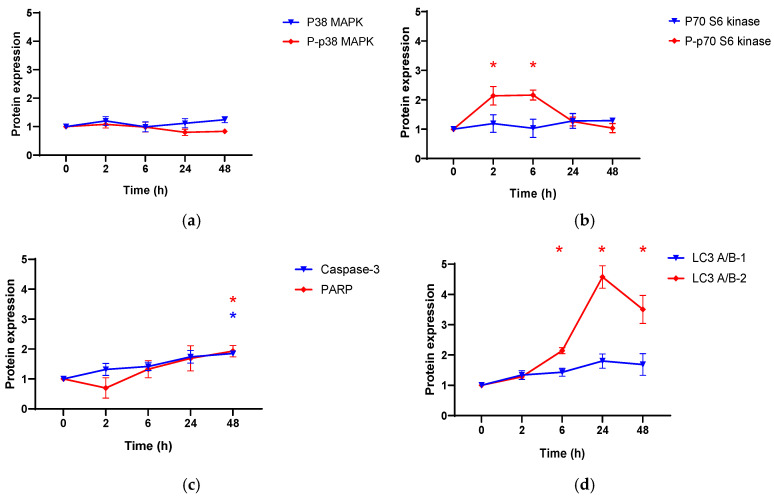
Effect of compound **4** on the expression of (**a**) p38 MAPK, p-p38 MAPK, (**b**) p70 S6 kinase, p-p70 S6 kinase, (**c**) caspase-3, PARP and (**d**) LC3 A/B-1, LC3 A/B-2 in PC3 cells. Each protein expression was normalized based on the expression of α/β-tubulin. Mean value and the standard deviation of the normalized value of three independent biological replicates is represented. * *p* < 0.05 compared to the zero-time point.

To support results obtained with flow cytometry, the effect of anthraquinone compound **4** on protein expression in PC3 cells was evaluated using western blot. As compound **4** is inducing apoptosis, as shown by flow cytometry, here specifically the influence on caspase 3 and PARP regulation in PC3 was assessed. Furthermore, the impact on p70 S6 kinase and p38 MAPK (mitogen-activated protein kinase) both non- and phosphorylated production upon treatment of PC3 cells with compound **4** was determined. The density of the tested protein bands was measured and compared to the density of housekeeping proteins (β-actin and α/β-tubulin), and protein expression relative to α/β-tubulin. Results are presented in Figure 6. Compound **4** caused the upregulation of caspase-3 and PARP after 48 h of treatment. Moreover, it induced early phosphorylation of P70 S6 kinase and the conversion of LC3 A/B-1 into LC3 A/B-2 till 24 h of treatment followed by downregulation as seen at the 48 h time point.

### 3.4. The Role of Autophagy

**Figure 7:**
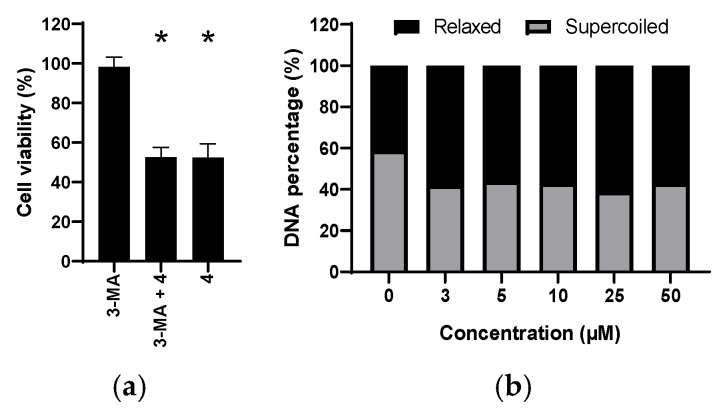
(**a**) Viability of PC3 cells treated with 3-MA and/or IC_50_ dose of compound **4** (CV assay, 48 h), * *p* < 0.05 compared to the 3-MA treated cells, no statistical significance between 3-MA + **4** and **4** treated cells; (**b**) the effect of compound **4** on the topoisomerase I activity in PC3 cells.

The effect of autophagy on cell death was studied by inhibiting the autophagy occurrence in PC3 cells and determining its impact on cell viability. The data obtained from the assay is illustrated in Figure 7a. The inhibition of autophagy by the use of 3-MA did not exert an impact on the viability of the PC3 cells when treated with compound **4**.

### 3.5. Influence of Compound 4 on the Topoisomerase Activity

Topoisomerase I is an enzyme which plays an important role in the DNA repair. It eliminates the pressure induced by the DNA supercoiling through introducing single strand break. The effect of compound **4** on topoisomerase I was tested. The compound caused a concentration independent increase in the activity of topoisomerase I. The percentage of relaxed DNA was increased by nearly 10% already at the lowest concentration as illustrated in Figure 7b.

## 4. Discussion

Most of the anthraquinone derivatives tested against PC3, HT-29, HeLa and HepG2, according to the National Cancer institute guidelines [29], must be considered anticancer agents as they possess IC_50_ values < 30 μg/mL. The cytotoxic effect of the active compounds was shown to be cell type specific in some cases. The most active compound against the PC3 cell line was derivative **4**, possessing an IC_50_ value of 4.65 µM.

The substitution pattern can significantly affect the anticancer activity of anthraquinone compounds. The structure-activity relationship for the investigated anthraquinones was evaluated for the PC3 cell line. Anthraquinone, the core structure of the investigated derivatives and its 1,4-dihydroxyl derivative (**30**), exhibit no cytotoxic activity on PC3 cells under the applied experimental settings (IC_50_ > 100 µM). Functionalization of anthraquinone by introducing hydroxyl groups at positions 1, 3 and 8 and methylation at position 6 (anthraquinone → emodin, a plant natural product) increases the anticancer potential to an IC_50_ of 30 µM (on PC3, 48 h).

The substitution pattern that enforces the best anticancer activity has an essential 1,4-*O*-disubstitution with lipophilic groups, e.g., two benzoxy or two *n*-butoxy groups. I.e., a motif with 1,4-dialkyloxy- or aralkyloxy-substitution is crucial while, interestingly 1,4-dihydroxyl substitution with its increased redox and tautomerism properties is inactive on PC3 and has little activity on other cell lines. The structure-activity relationship points out that the balanced lipophilicity of the compounds is essential for the in vitro activity. Moreover, the passage of biological membranes for these compounds might be facilitated, thus enabling access to intracellular biological targets. The most active compounds are those with lipophilic groups at position 1 and 4.

Compounds **1**, **4** and **16** share the same core motif (1,4-di-benzyloxyanthraquinone) but they differ by their substituents in positions 2 and 3. The presence of two hydroxymethyl groups (compound **4**) in positions 2 and 3 is essential for their improved activity. The modification of these groups by ring forming protection as acetonide (compound **1**) slightly reduced the anticancer activity (1.5-fold). However, the presence of only butyryl at position 2 and no substitution at position 3 (compound **16**) significantly reduced the activity by fivefold. By comparing the structures of compounds **21** and **22**, the replacement of a 2-keto by its reduced 2-(1-hydroxyalkyl) group, i.e., of propionyl by a reduced but similar 1-hydroxy-butyl, increased the activity 3-fold.

Obviously, inhibition of a para-dihydroxy (or similar easily oxidized) pre-quinoid constellation has a positive influence. Another way to avoid this is omission of one of the hydroxyl or alkoxy groups. Therefore, compounds with just one hydroxyl group were tested and found to have an activity similar to some of the 1,4-dialkoxy derivatives. The substitution of the functional groups on the side chains of the 1-hydroxyanthraquinone tested did not show significant changes in the compounds’ anticancer activity. The 2-hydroxymethylderivative **31** did not impact PC3 cells growth till 100 µM. Protecting the hydroxyl of the 2-hydroxylmethyl with a benzoyl group (compound **18**) results in a dramatic increase of activity. This activity can be modulated by different acid components, e.g., 4-nitrobenzoate **19** or cinnamate **20** cause a decrease and an increase, respectively, of activity although on moderate scale. The positive effect of avoiding *o*- or *p*-hydroquinoid patterns (cf. inactive compound **31**) in the A- or C-ring of anthraquinones is corroborated by the 1,3-dihydroxylderivative **33**, which shows cytotoxicity again (ca. < 30 µM on PC3, and 6 µM on HeLa).

Compound **4** showed an activity (IC_50_ = 4.65 µM) surpassing that of the natural anticancer compound emodin (IC_50_ = 30 µM) six-fold. However, on this specific cell line which was in the focus of the work presented herein, both compounds only induced cell death. It is well known that emodin may induce differentiation of various cancers (e.g., acute myeloid leukemia cancer cells NB4) to normal cells [24]. The apoptosis induced by compound **4** could be linked to activation of the caspase pathway. Toxic metabolite generation such as ROS/RNS and NO may be involved in the regulation of the caspase expression, therefore, DHR and DAF-FM assays were performed. Compound **4** caused the reduction of both ROS/RNS and NO. This can be explained because anthraquinones can act as free radical scavengers and possess the ability to trap oxygen and interfere with redox and hydrogen peroxide signaling and action [61,62]. Although, autophagy on first sight appears not to be increased significantly, as autophagy vesicles are elevated only minimally. However, further analysis with western blot clearly showed the formation of the LC3A/B-II fragment, a highlight of autophagy. The low autophagy upon 48 h treatment of PC3 cells with compound **4** detected in the AO assay is in good agreement with expression of the LC3A/B-II fragment after the same time of exposure. However, a peak of LC3A/B-II fragment expression was detected at 24 h. Consequently, additional experiments, in which the role of autophagy on the mechanism of action for the PC3 cells, namely cytoprotective or cytotoxic impact, was performed. The autophagy occurrence in PC3 cells was inhibited using 3-MA. The inhibition of autophagy did not result in any restoration of PC3 cell viability when treated with compound **4**. Therefore, the occurrence of autophagy is a mode adapted by the PC3 cells for cytoprotective causes.

P70 S6 kinase is a ribosomal protein which plays an essential role in the regulation of protein expression through the phosphorylation 40S ribosomal subunit of protein S6 [63] which causes the induction of mRNA translation and protein synthesis [64]. Compound **4** shows cytotoxic effects on PC3 cells by trapping the cells in the G2/M phase, the phase in which protein synthesis takes place. This explains the overexpression of P-p70 S6 kinase. The cytotoxic effect of the compound was confirmed by a cell division assay where only an insignificant influence on cell division was detected. On the contrary, compound **4** did not impact neither the phosphorylation nor the expression of P38 MAPK, one of the main members of the MAPK pathway. This pathway is key regulator of apoptosis through controlling the release of the cytokines by the immunity system as a response to stress stimuli [65].

PARP is a protein expressed in the nuclei of most mammalian cells. It is involved in DNA repair. When DNA is exposed to damaging agents such as alkylating agents or radiation, PARP binds to SSB and acts as a signal for DNA repairing enzymes [53]. Compound **4** caused an increase in the PARP expression level in PC3 cells. Consequently, as a response to the induced DNA damage, the biosynthesis of PARP increased. For the proper functioning of PARP, the DNA must be unfolded by the effect of topoI. This assumption was further investigated by studying the effect of compound **4** on topoI which was found to enhance topoI activity.

## 5. Conclusions

In vitro determination of the anticancer activity of anthraquinone compounds against four different cell lines PC3, HT-29, HeLa and HepG2 was the main goal of this work. 33 different compounds were tested to determine their cytotoxicity against PC3, HT-29, HeLa and HepG2 cell lines and most of the compounds exhibit a moderate cytotoxic effect. The IC_50_ values of most compounds were determined and ranged from 4.65–>100 µM. Some anthraquinone derivatives turned out to be tumor cell type-specific agents, with derivative compound **4** showing the most prominent effect on prostate cancer (IC_50_ = 4.65 µM, PC3 cell line, 48 h).

The limited number of derivatives allowed only for a very basic SAR. But already this small set demonstrated that additional hydroxyl and especially alkoxy groups at the anthraquinone core are required for any activity. Free hydroquinonemoieties within the anthraquinone appear to be detrimental, although data are not yet sufficient for a definite conclusion. However, none of the commercial or natural derivatives with anticancer activity has such a para or ortho hydroquinol structure—likely because of redox effects. In protected form, e.g., as 1,4-*O*-alkyl groups, activity is restored or increased. In any case, 2- and/or 3-(1-hydroxyalkyl) substitutions are beneficial and can be used to fine tune activity. If these effects are related to electronic or steric influences, to molecular function or simply improved cell bioavailability and targeting cannot be differentiated at this time.

The mode of action of compound **4** against the PC3 cell line involves cytotoxicity induced by apoptosis. The apoptosis is accompanied by the activation of the caspase pathways. Overexpression of the P-p70 S6 kinase (necessary for protein biosynthesis) may be correlated to the observed strong accumulation of the cells in the G2/M phase, which might indicate that cell cycle arrest most probably occurs in the G2 phase (protein biosynthesis). Moreover, the role of autophagy in the mode of cell death of PC3 cells was rolled out. On the other hand, a differentiation of cell types speaks more for a redox signaling intervention, as the different cell lines respond differently to interferences in this system, including the famous Warburg effect or the distinct effect of selenium compounds on prostate and colon cancer cells [61,66,67].

## Supplementary Materials

The following information is available online at https://www.mdpi.com/article/10.3390/cells11010168/s1, Figure S1: PC3 cell viability after 48 h treatment with anthraquinone derivatives (* Interfering with MTT assay), Figure S2: HT-29 cell viability after 48 h treatment with anthraquinone derivatives (* Interfering with MTT assay), Figure S3: HeLa cell viability after 48 h treatment with anthraquinone derivatives (* Interfering with MTT assay), Figure S4: HepG2 cell viability after 48 h treatment with anthraquinone derivatives (* Interfering with MTT assay), Figure S5: Dose-dependent response of PC3 cells treated with anthraquinone derivatives determined by MTT assay (48 h), Figure S6: Dose-dependent response of PC3 cells treated with anthraquinone derivatives determined by CV assay (48 h), Figure S7: Dose-dependent response of HT-29 cells treated with anthraquinone derivatives determined by MTT assay (48 h), Figure S8: Dose-dependent response of HT-29 cells treated with anthraquinone derivatives determined by CV assay (48 h), Figure S9: Dose-dependent response of HeLa cells treated with anthraquinone derivatives determined by MTT assay (48 h), Figure S10: Dose-dependent response of HeLa cells treated with anthraquinone derivatives determined by CV assay (48 h), Figure S11: Dose-dependent response of HepG2 cells treated with anthraquinone derivatives determined by MTT assay (48 h), Figure S12: Dose-dependent response of HepG2 cells treated with anthraquinone derivatives determined by CV assay (48 h), Table S1: Structures and IC_50_ values (µM) of anthraquinones tested, determined by MTT and CV assay against HT-29, HeLa and HepG2 cell lines (48 h of treatment).
