# Supplementary material for: In Vitro Anticancer Screening and Preliminary Mechanistic Study of A-Ring Substituted Anthraquinone Derivatives
Source: Cells. 2022 Jan 5;11(1):168. doi: 10.3390/cells11010168 (PMC8750254; doi:10.3390/cells11010168)
Supplement: Supplementary file 1 [file cells-11-00168-s001.zip › cells-1519142-supplementary.pdf]

# *In vitro* anticancer screening and preliminary mechanistic study of A-ring substituted anthraquinone derivatives

**Ibrahim Morgan [1], Ludger A. Wessjohann [1,*] and Goran N. Kaluđerović [1,2,*]**

[1] Department of Bioorganic Chemistry, Leibniz Institute of Plant Biochemistry, Weinberg 3, 06120, Halle (Saale), Germany; ibrahim.morgan@ipb-halle.de; wessjohann@ipb-halle.de; goran.kaluderovic@ipb-halle.de

[2] Department of Engineering and Natural Sciences, University of Applied Sciences Merseburg, Eberhard-Leibnitz-Straße 2, 06217 Merseburg, Germany; goran.kaluderovic@ hs-merseburg.de

* Correspondence: goran.kaluderovic@hs-merseburg.de (ORCID 0000-0001-5168-1000); wessjohann@ipb-halle.de (ORCID 0000-0003-2060-8235)

## Supporting Information

### 1. Structures and $IC_{50}$ values of anthraquinone derivatives

**Table S1:** Structures and $IC_{50}$ values (μM) of anthraquinones tested, determined by MTT and CV assay against HT-29, HeLa and HepG2 cell lines (48 h of treatment)

| **Compound no.** | **Structure** | **PC3** | | **HT-29** | | **HeLa** | | **HepG2** | |
|---|---|---|---|---|---|---|---|---|---|
| | | **$IC_{50}$ [μM]** | | | | | | | |
| | | **MTT** | **CV** | **MTT** | **CV** | **MTT** | **CV** | **MTT** | **CV** |
| **1** | 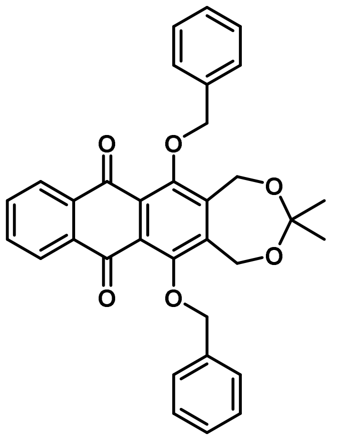 | 7.38±0.66 | 7.30±0.53 | 7.78±0.23 | 13.93±0.26 | 7.80±0.27 | 11.94±1.57 | 5.65±0.89 | 7.46±1.37 |

| | | | | | | | | | |
|---|---|---|---|---|---|---|---|---|---|
| **2** | 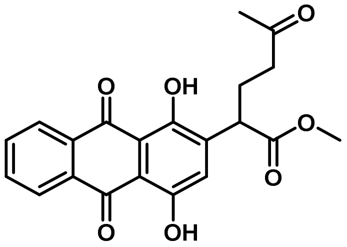 | 37.50±0.35 | 49.51±5.43 | >100 | 47.04±4.33 | 70.12±0.12 | 26.86±1.73 | 8.97±0.93 | 26.40±0.15 |
| **3** | 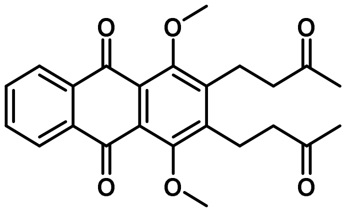 | 35.13±0.20 | 41.17±0.12 | 15.72±0.62 | 25.80±2.79 | 8.55±1.48 | 11.38±0.68 | 6.67±3.07 | 18.23±2.89 |
| **4** | 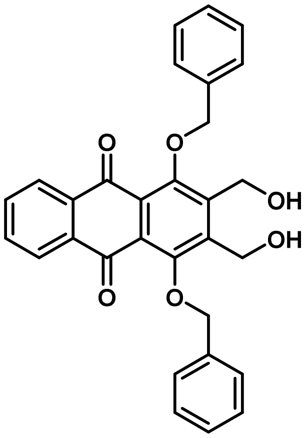 | 6.89±0.83 | 4.65±0.67 | 4.58±0.99 | 6.97±1.08 | 4.85±0.80 | 6.17±0.89 | 4.53±0.56 | 5.50±0.42 |
| **5** | 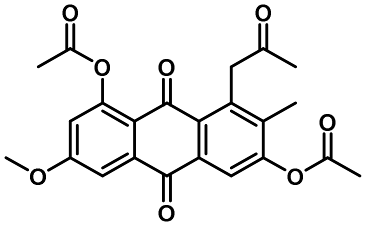 | 11.67±1.17 | 17.79±2.90 | 22.72±3.67 | 26.36±1.73 | 5.14±0.66 | 6.92±0.40 | 3.44±1.07 | 6.39±1.95 |
| **6** | 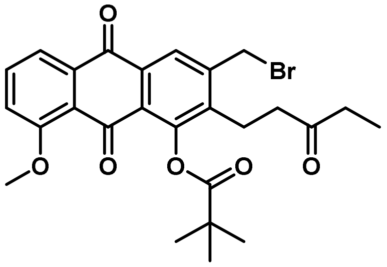 | 5.50±0.58 | 6.59±0.37 | 8.72±0.49 | 9.86±0.38 | 6.84±0.43 | 6.50±1.17 | 6.14±1.05 | 6.26±1.52 |
| **7** | 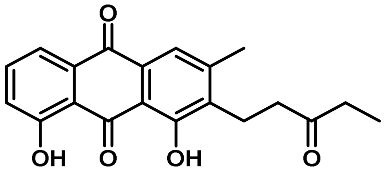 | >100 | >100 | >100 | >100 | >100 | >100 | >100 | >100 |

| | | | | | | | | | |
|---|---|---|---|---|---|---|---|---|---|
| **8** | 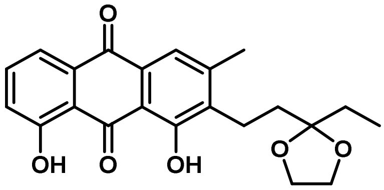 | >100 | >100 | >100 | >100 | >100 | >100 | >100 | >100 |
| **9** | 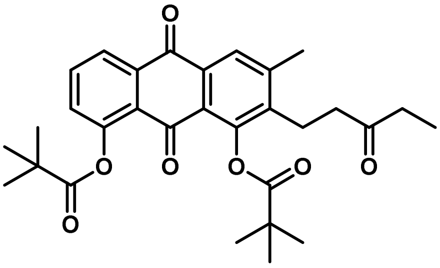 | 10.58±1.04 | 10.98±1.10 | 20.95±2.96 | 32.06±3.29 | 7.81±0.49 | 15.11±0.85 | 4.57±0.73 | 9.94±1.73 |
| **10** | 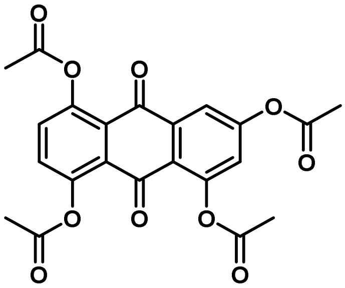 | 32.03±3.65 | 36.14±2.37 | 25.04±3.73 | 33.32±1.09 | 14.53±0.26 | 23.32±1.58 | 13.04±0.74 | 29.78±4.47 |
| **11** | 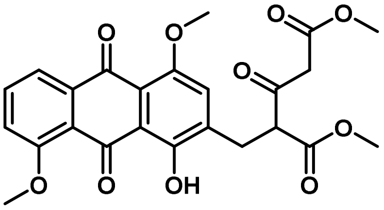 | >100 | >100 | >100 | >100 | >100 | >100 | >100 | 36.14±1.31 |
| **12** | 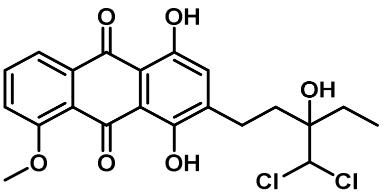 | 23.75±1.28 | 14.16±2.10 | 9.29±0.88 | 20.26±1.13 | 14.75±1.78 | 15.05±2.23 | 21.75±0.70 | 2.03±2.22 |
| **13** | 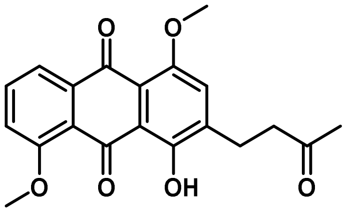 | 26.27±2.79 | 30.23±3.10 | >100 | 38.14±1.87 | 9.51±0.65 | 12.84±0.98 | 7.24±1.19 | 14.27±1.26 |

| | | | | | | | | | |
|---|---|---|---|---|---|---|---|---|---|
| **14** | 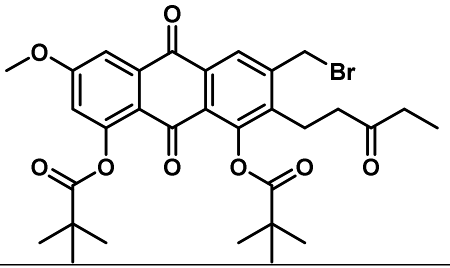 | 8.94±1.52 | 8.52±0.47 | 9.29±1.53 | 18.78±1.74 | 6.18±0.56 | 8.42±0.08 | 11.65±0.79 | 12.36±0.95 |
| **15** | 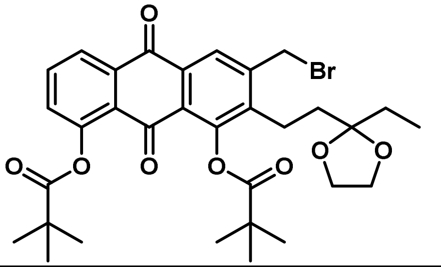 | 15.18±1.15 | 14.71±1.33 | 31.19±1.64 | 28.23±1.78 | 11.23±0.68 | 14.16±0.31 | 12.03±0.82 | 24.19±2.51 |
| **16** | 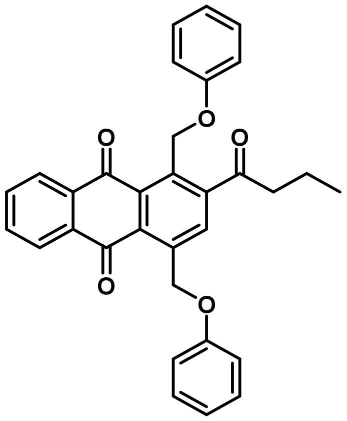 | 19.82±1.03 | 24.62±2.18 | >100 | >100 | 14.66±0.02 | 44.92±3.26 | 6.82±0.36 | 10.20±0.89 |
| **17** | 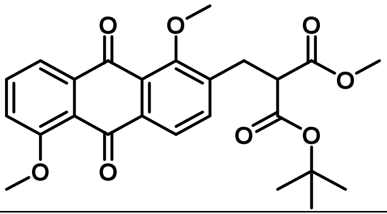 | 30.99±2.58 | 29.00±4.58 | 13.37±0.59 | 30.56±0.40 | 29.23±2.29 | 35.19±2.24 | 5.98±0.37 | 20.07±0.11 |
| **18** | 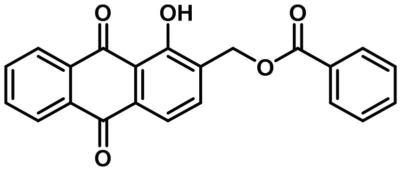 | 13.94±1.44 | 18.79±1.76 | 4.72±0.33 | 21.37±1.87 | 8.35±1.32 | 9.58±0.73 | 10.57±0.33 | 25.26±3-26 |
| **19** | 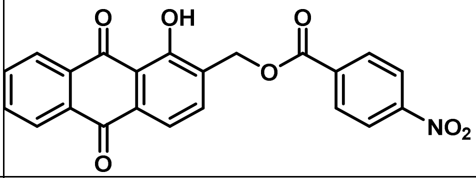 | 31.71±1.22 | 27.28±2.96 | 40.35±1.57 | 37.32±1.68 | 22.49±1.21 | 14.26±1.42 | 15.06±0.73 | 14.94±1.76 |
| **20** | 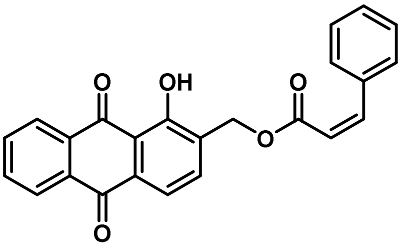 | 10.15±1.73 | 12.97±0.41 | 2.52±0.67 | 16.72±1.41 | 7.84±1.75 | 9.63±1.23 | 6.08±1.82 | 9.90±1.98 |

| | | | | | | | | | |
|---|---|---|---|---|---|---|---|---|---|
| **21** | 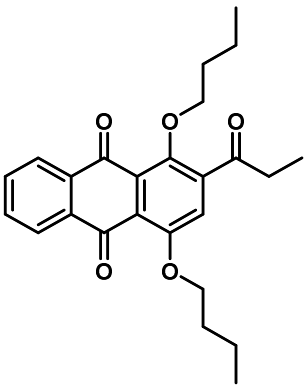 | 26.29±1.73 | 30.00±2.28 | >100 | >100 | 41.31±3.37 | 95.23±5.37 | 9.32±0.92 | 17.79±0.35 |
| **22** | 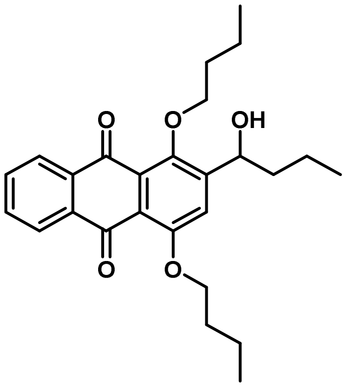 | 8.48±0.10 | 11.10±0.83 | 6.70±0.09 | 10.35±0.48 | 4.18±0.09 | 8.20±0.18 | 3.14±0.34 | 4.60±0.53 |
| **23** | 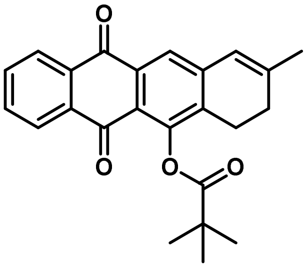 | >100 | >100 | >100 | >100 | >100 | >100 | 45.76±8.24 | 33.25±3.37 |
| **24** | 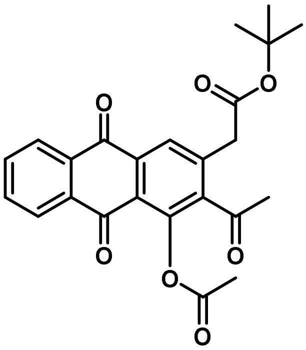 | >100 | 77.41±6.15 | >100 | >100 | >100 | 67.10±4.21 | >100 | 35.77±0.94 |
| **25** | 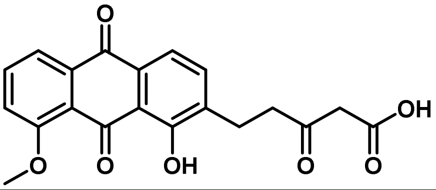 | 31.42±3.23 | 36.74±2.71 | 3.95±0.19 | 19.61±1.69 | 5.13±0.21 | 12.94±1.26 | 11.82±1.41 | 10.65±1.33 |

| | | | | | | | | | |
|---|---|---|---|---|---|---|---|---|---|
| **26** | 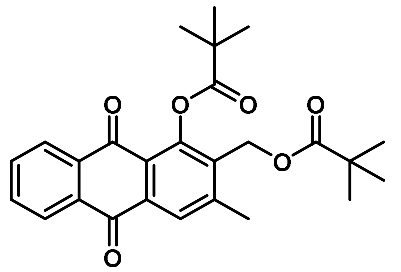 | 23.48±1.19 | 28.12±1.81 | 15.64±1.39 | 24.09±2.16 | 22.51±1.78 | 27.12±1.88 | 27.78±1.93 | 9.54±1.03 |
| **27** | 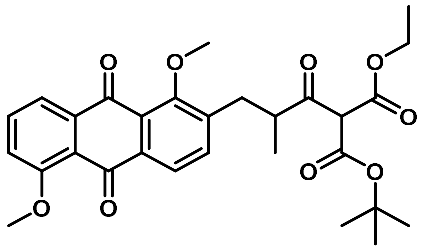 | 45.84±3.23 | 38.77±3.82 | 80.71±4.19 | 94.11±1.59 | 60.34±1.72 | 67.18±3.55 | 5.48±0.21 | >100 |
| **28** | 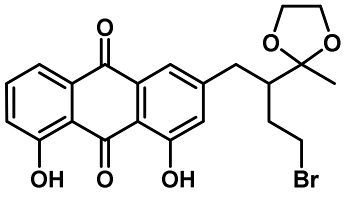 | 16.50±1.86 | 22.05±2.95 | 10.50±1.86 | 27.49±1.79 | 12.12±1.45 | 20.00±2.51 | 6.31±0.35 | 30.75±3.40 |
| **29** | 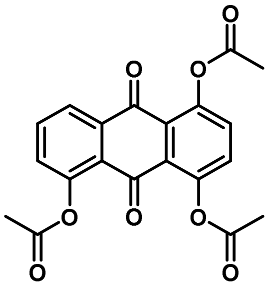 | * | 10.89±1.87 | * | 32.22±1.69 | * | 13.05±1.21 | * | 3.81±0.57 |
| **30** | 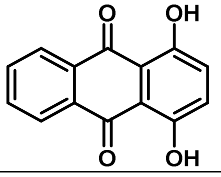 | >100 | >100 | 21.24±0.67 | 41.45±3.82 | 20.64±1.11 | 30.56±0.43 | 13.34±0.22 | 38.29±2.99 |
| **31** | 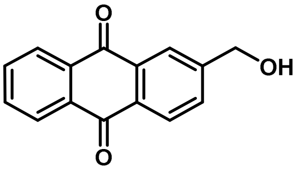 | >100 | >100 | 39.13±2.83 | 54.86±4.74 | 10.65±0.60 | 13.75±1.72 | 53.32±4.10 | 62.52±2.21 |
| **32** | 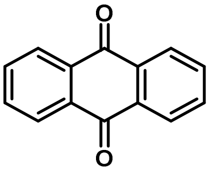 | >100 | >100 | >100 | >100 | 83.13±1.32 | 80.72±1.68 | >100 | >100 |
| **33** | 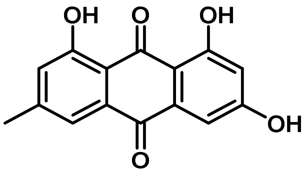 | 22.01±1.34 | 30.07±2.66 | 43.76±1.64 | 41.70±1.62 | 5.95±0.96 | 7.36±0.62 | 20.63±2.92 | 23.50±1.22 |

*Interfering with MTT assay

## 2. Fast screening results

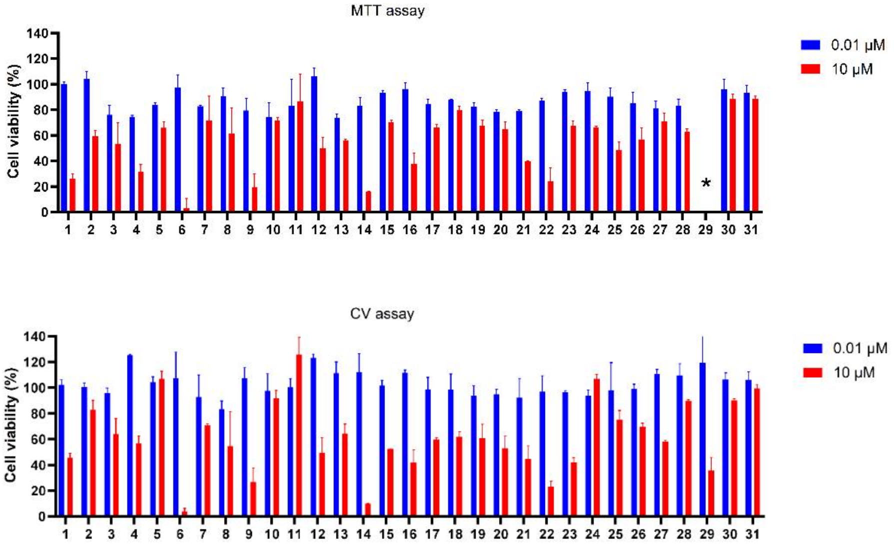


**Figure S1:** PC3 cell viability after 48 h treatment with anthraquinone derivatives (*Interfering with MTT assay)

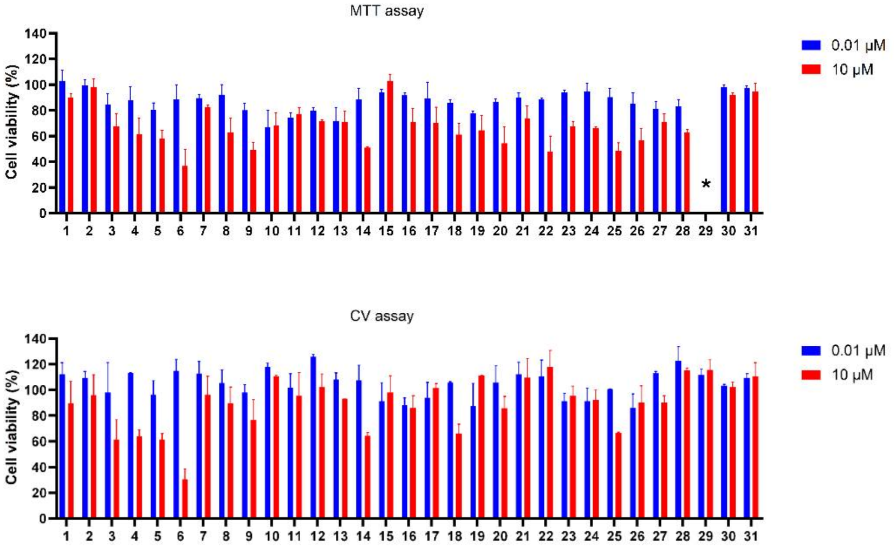


**Figure S2:** HT-29 cell viability after 48 h treatment with anthraquinone derivatives (*Interfering with MTT assay)

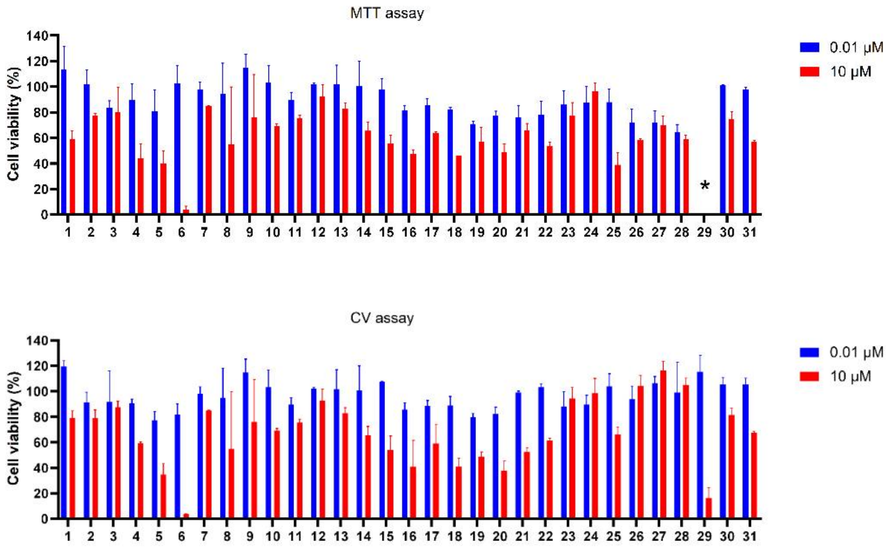


**Figure S3:** HeLa cell viability after 48 h treatment with anthraquinone derivatives (*Interfering with MTT assay)

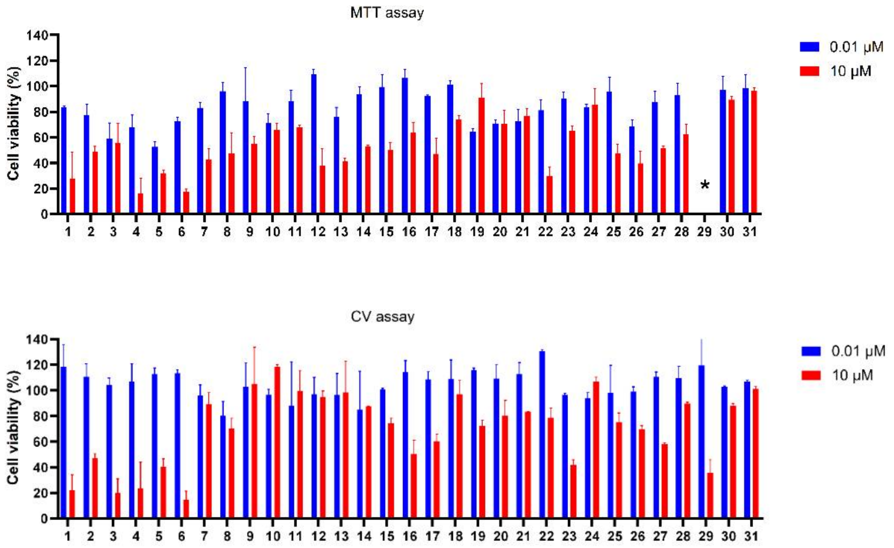


**Figure S4:** HepG2 cell viability after 48 h treatment with anthraquinone derivatives (*Interfering with MTT assay)

## 3. IC₅₀ determination

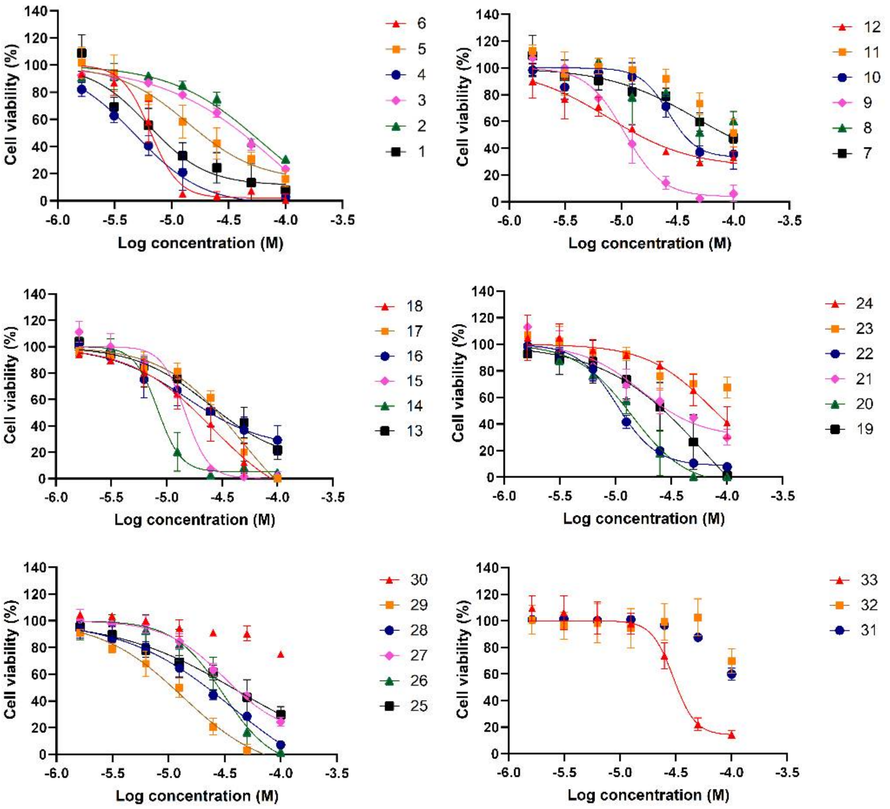


**Figure S5:** Dose-dependent response of PC3 cells treated with anthraquinone derivatives determined by MTT assay (48 h)

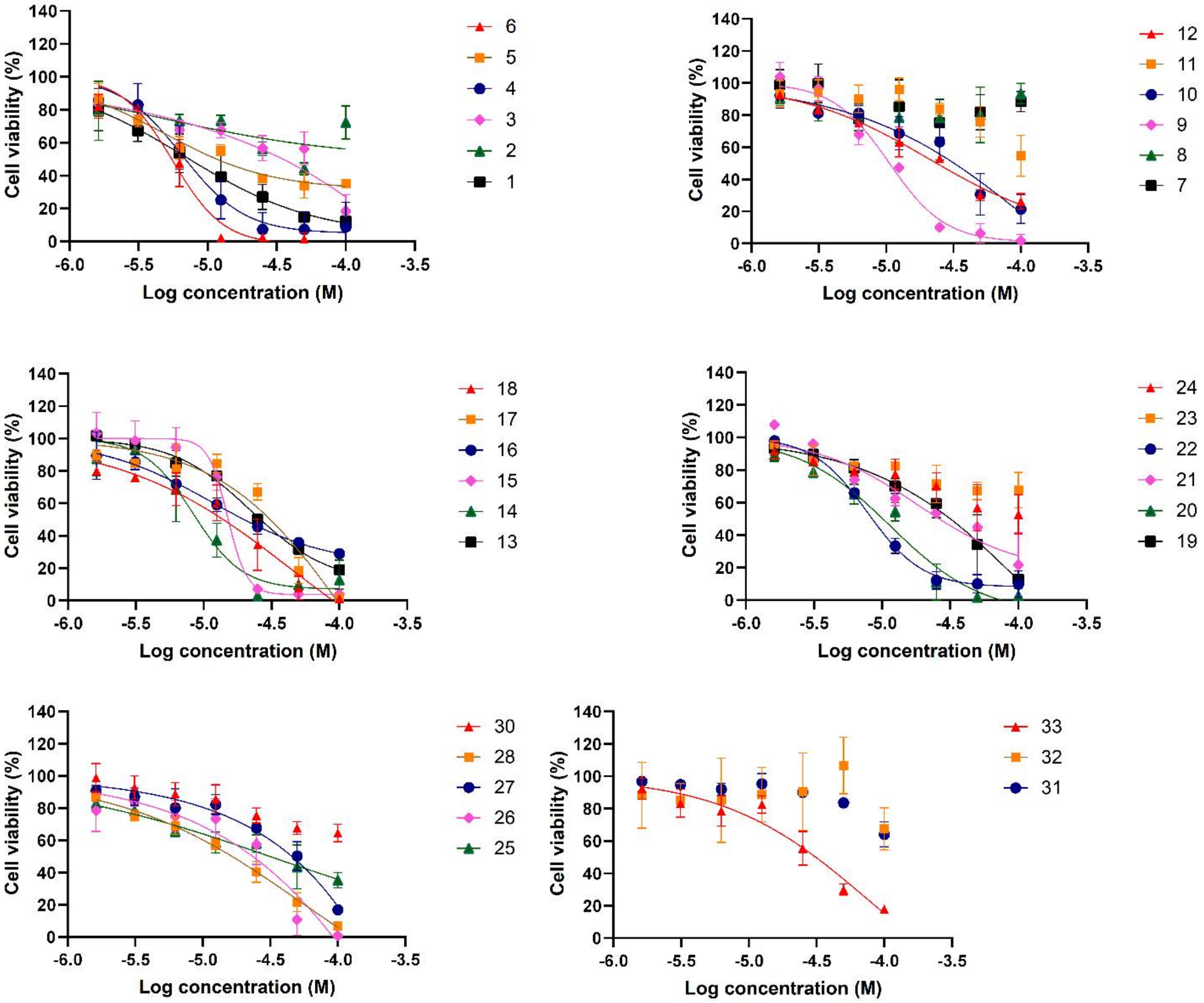


**Figure S6:** Dose-dependent response of PC3 cells treated with anthraquinone derivatives determined by CV assay (48 h)

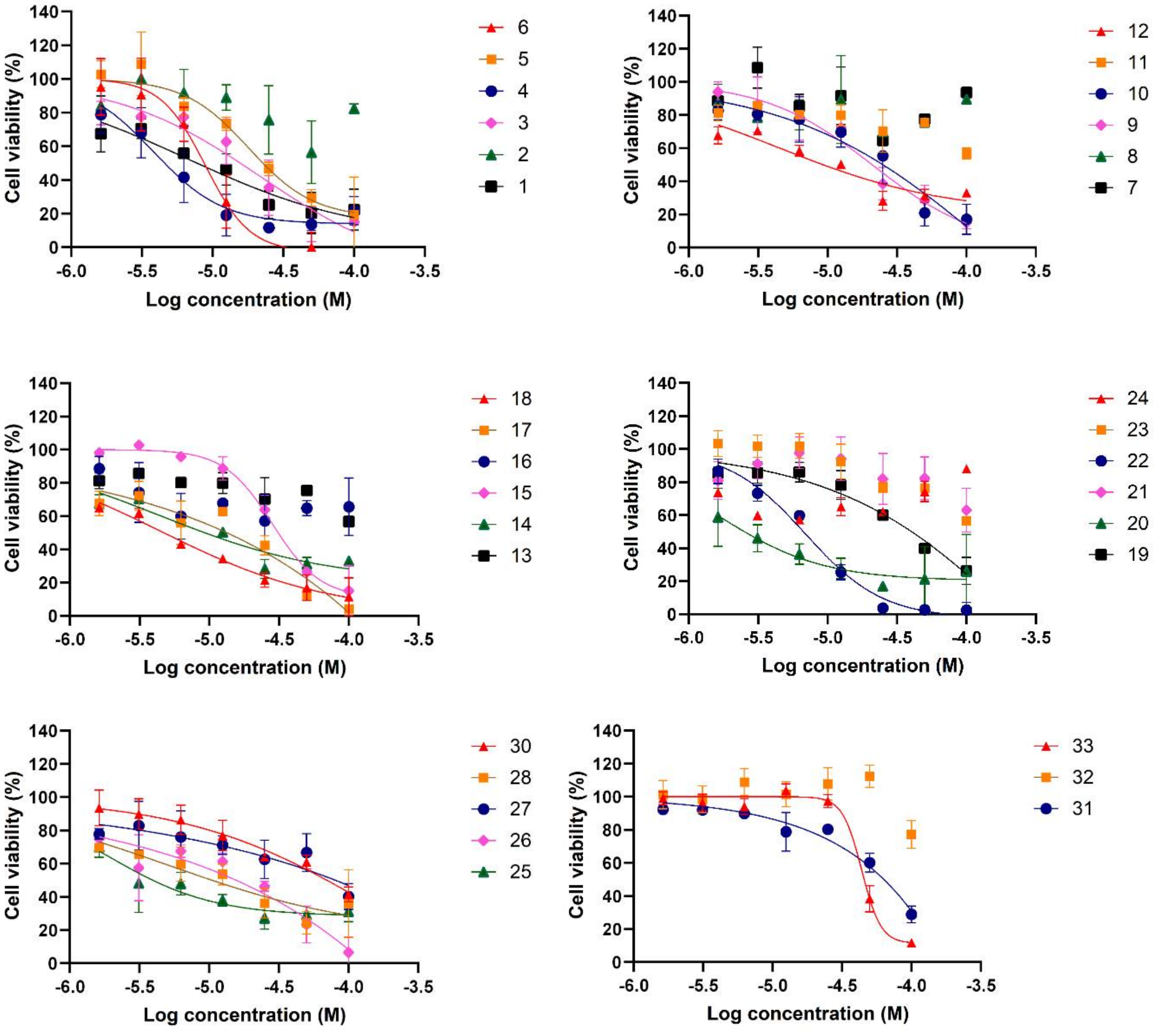


**Figure S7:** Dose-dependent response of HT-29 cells treated with anthraquinone derivatives determined by MTT assay (48 h)

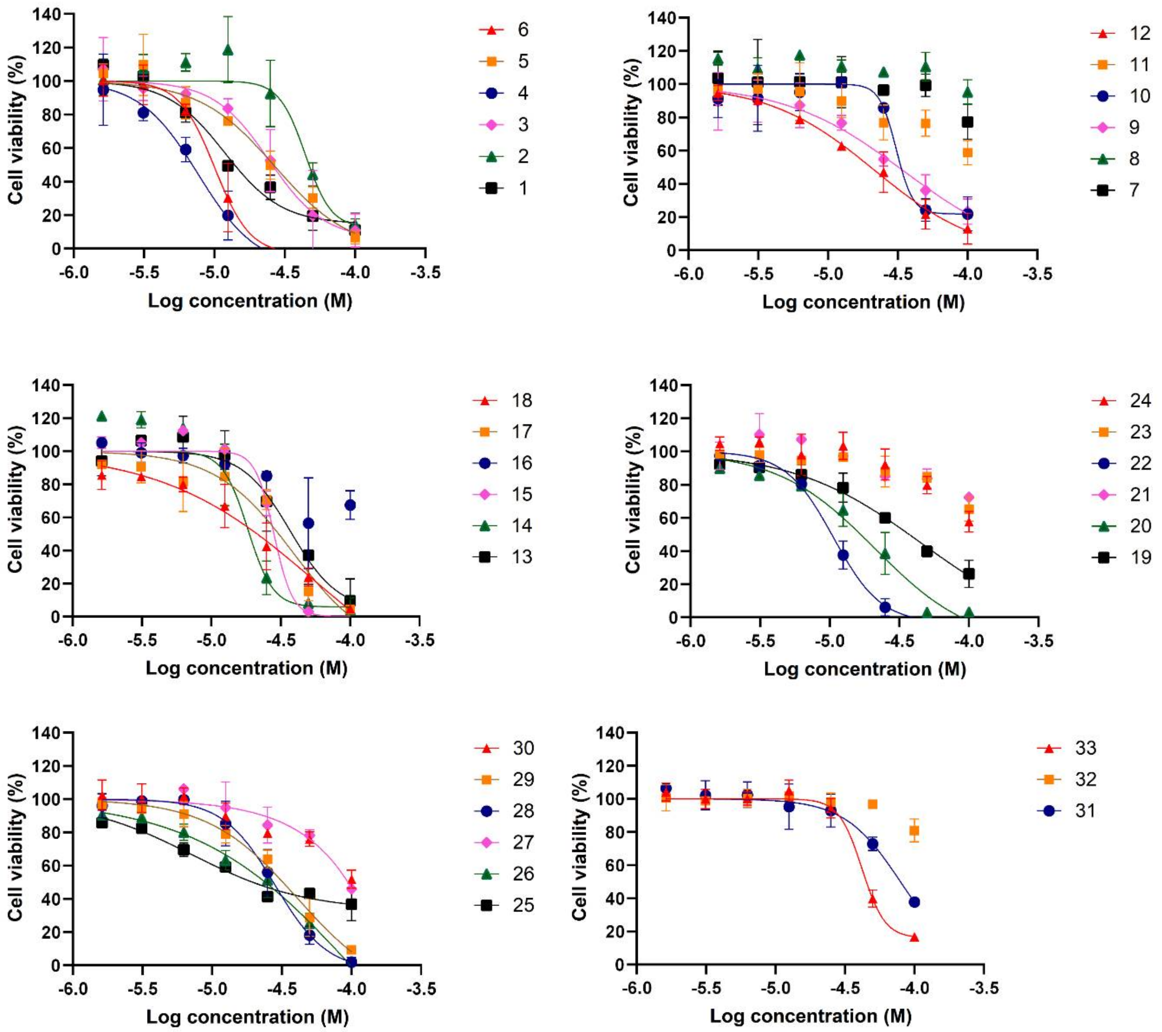


**Figure S8:** Dose-dependent response of HT-29 cells treated with anthraquinone derivatives determined by CV assay (48 h)

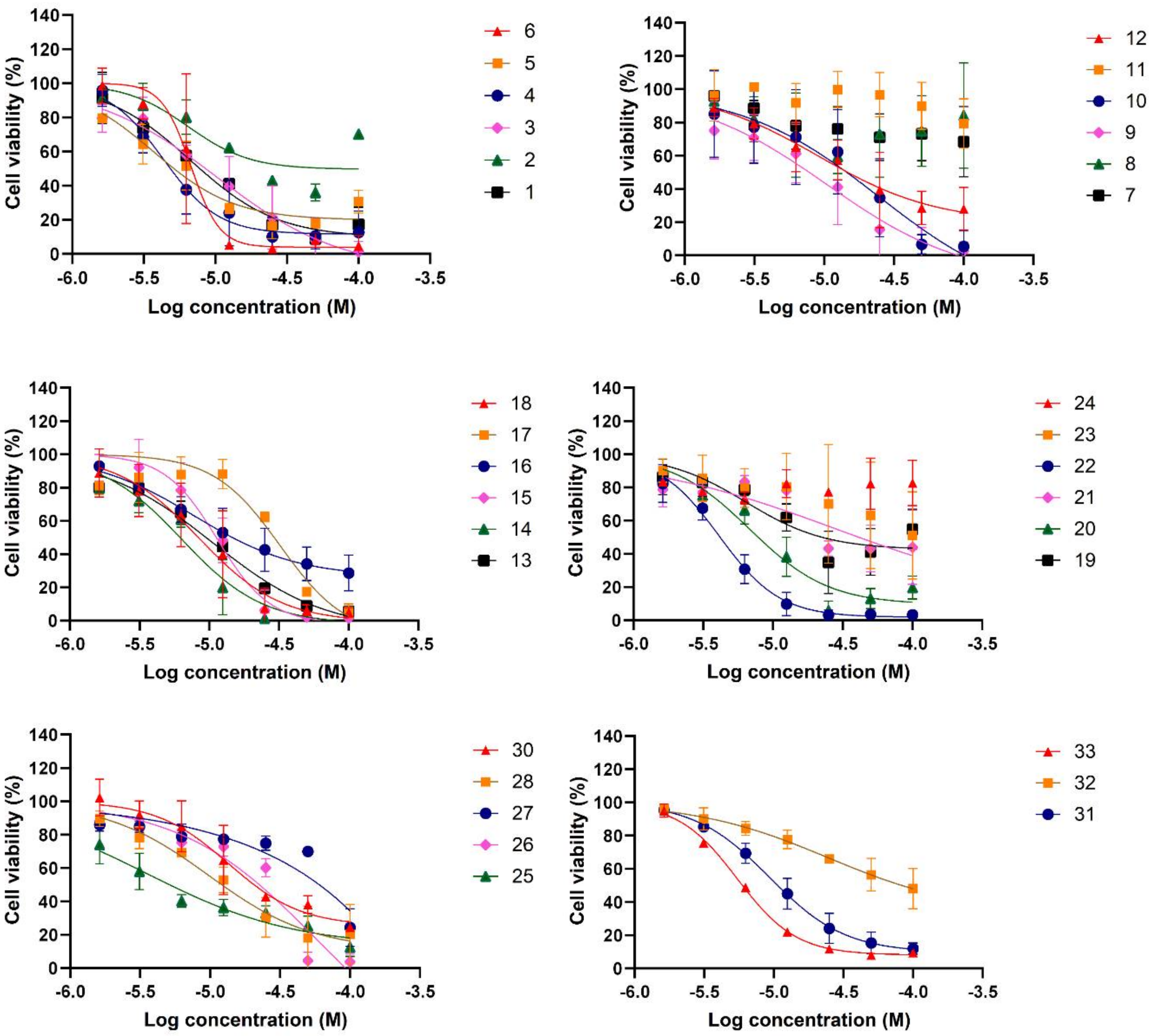


**Figure S9:** Dose-dependent response of HeLa cells treated with anthraquinone derivatives determined by MTT assay (48 h)

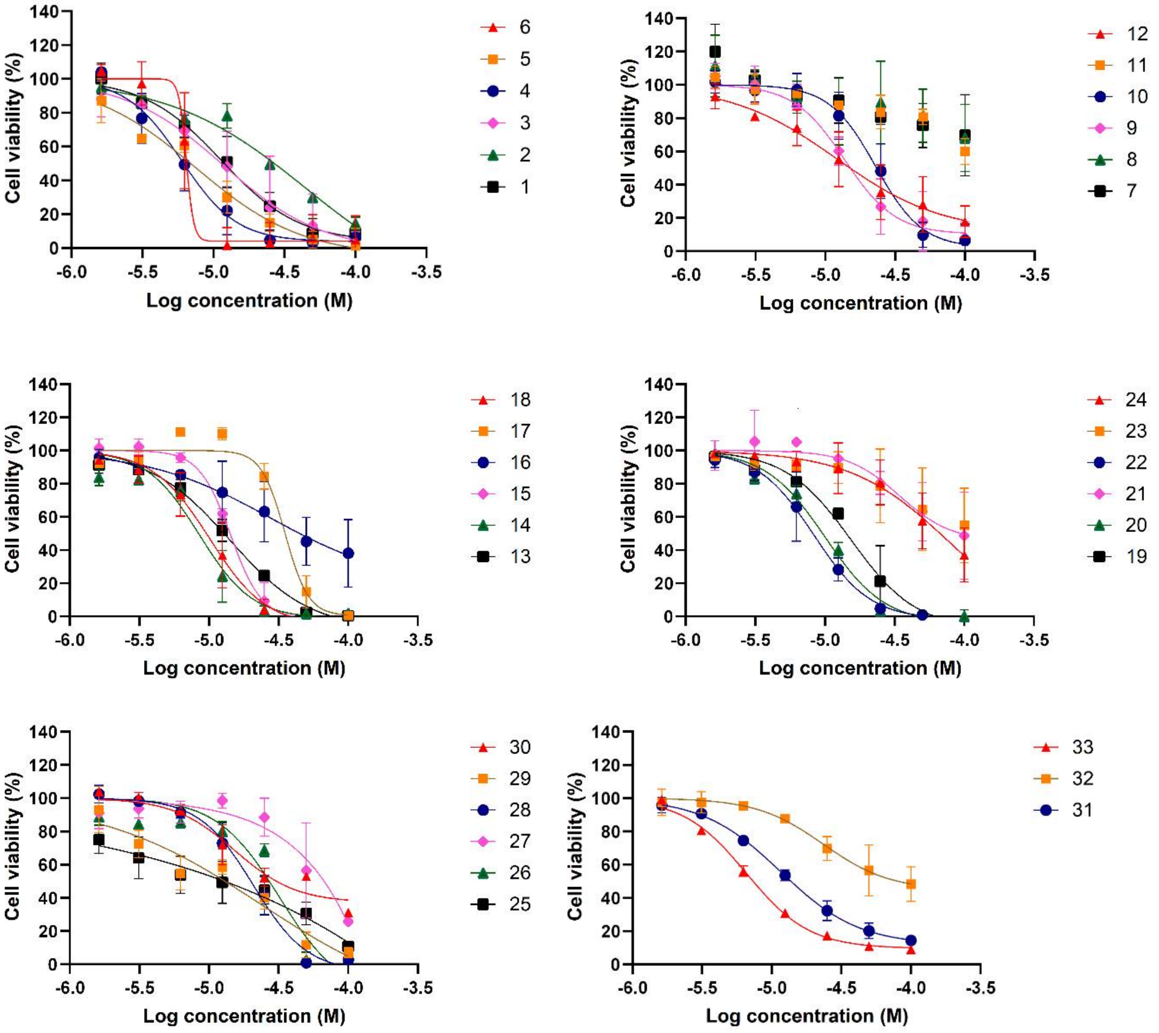


**Figure S10:** Dose-dependent response of HeLa cells treated with anthraquinone derivatives determined by CV assay (48 h)

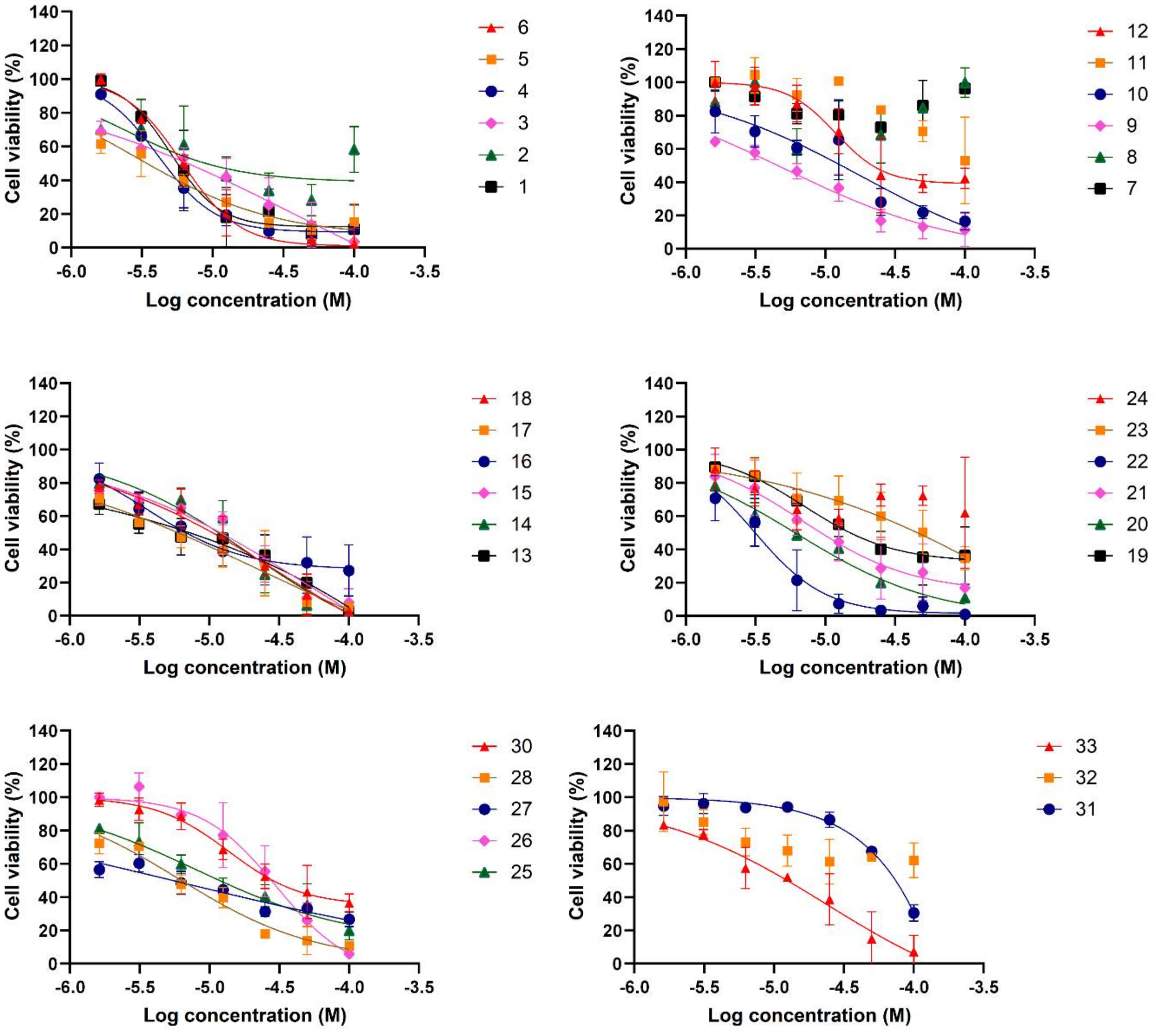


**Figure S11:** Dose-dependent response of HepG2 cells treated with anthraquinone derivatives determined by MTT assay (48 h)

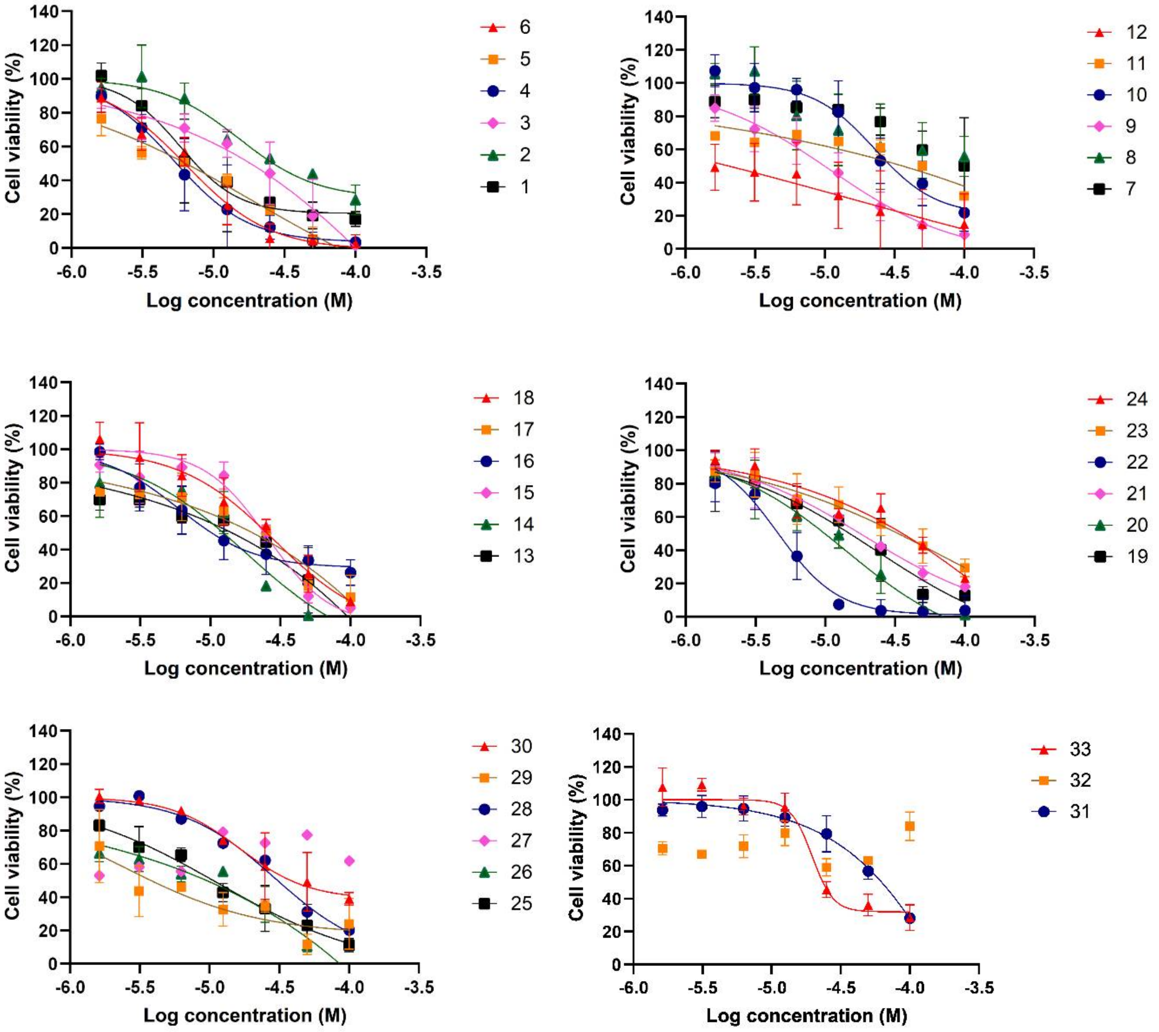


**Figure S12**: Dose-dependent response of HepG2 cells treated with anthraquinone derivatives determined by CV assay (48 h)
